# 

**Published:** 1848-05

**Authors:** 


### CONTENTS

OF NO. V.

### ORIGINAL COMMUNICATIONS.

### ESSAYS AND CASES.

Art. I.—The Family of Araneides, or Spiders—their Natural History—the Effects and Treatment of their Bite—and the Use of their Web as an Article of the Materia Medica. By Charles A. Hentz, M.D., of Alabama. (Being an Inaugural Dissertation, submitted to the Trustees and Medical Faculty of the University of Louisville, February 1848, for the Degree of M.D.)............369

Art. II.—A Case of Cæsarian Section; rendered necessary by the presence of a Tumor in the Vagina. By W. H. Byford, M. D., of Mt. Vernon, Ind....................383

Art. III.—Notes on Medical Matters and Medical Men in Paris. By David W. Yandell, M. D., of Lousville, Kentucky..................................................387

### REVIEWS.

Art. IV.—The Practice of Medicince: A Treatise on Special Pathology and Therapeutics. By Robley Dunglison, M.D., Professor of the Institutes of Medicine, &c., in the Jefferson Medical College, Philadelphia; Lecturer on Clinical Medicine, etc.

A Treatise on the Practice of Medicine. By George B. Wood, M. D., Professor of Materia Medica and Pharmacy in the University of Pennsylvania; one of the authors of the Dispensatory of the United States of America, etc..............................................404

SELECTIONS FROM AMERICAN AND FOREIGN JOURNALS.

Treatment of Dyspeptic Headache....441
On the Treatment of Fever by Cold Water....443
Discharge of a Tooth from the Ear....448
Treatment of Epilepsy....449
On the Internal use of Nitrate of Silver in Obstinate Diarrhea and Dysentery....450
M. Kun's New Instrument for the Diagnosis of Tumors....452

ORIGINAL INTELLIGENCE.

Medical Commencement....453
Medical Classes....453
Death of Professor Randolph....454
Faculty of Medicine of Paris....454
Subsidence of Cholera....454
Progress of the Use of Chloroform....455
Introductory Lectures....456
Solly on the Human Brain....457
Disorders of the Cerebral Circulation....457
Bloomingdale Asylum....457
Transactions of the Philadelphia College of Physicians....458
Churchill's Midwifery....458
Whitehead on Abortion and Sterility....458
Taylor on Poisons....459
Matteucci on Living Beings....459
Identities of Light, Heat, and Electricity....459
Resignation of Dr. Bayless....460
American Medical Association....461
Epidemic Scarlatina....461

